# Transcription Kinetics in the Coronavirus Life Cycle

**DOI:** 10.1002/wrna.70000

**Published:** 2025-01-05

**Authors:** Katarzyna Grelewska‐Nowotko, Ahmed Eisa Elhag, Tomasz Wojciech Turowski

**Affiliations:** ^1^ Institute of Biochemistry and Biophysics Polish Academy of Sciences Warsaw Poland; ^2^ Department of Preventive Medicine and Clinical Studies, Faculty of Veterinary Sciences University of Gadarif Al Qadarif Sudan

**Keywords:** coronaviruses, discontinuous transcription, RNA‐dependent RNA polymerase, RNA–RNA interactions, ssRNA(+) viruses, subgenomic RNA, transcription, viral genome replication

## Abstract

Coronaviruses utilize a positive‐sense single‐strand RNA, functioning simultaneously as mRNA and the genome. An RNA‐dependent RNA polymerase (RdRP) plays a dual role in transcribing genes and replicating the genome, making RdRP a critical target in therapies against coronaviruses. This review explores recent advancements in understanding the coronavirus transcription machinery, discusses it within virus infection context, and incorporates kinetic considerations on RdRP activity. We also address steric limitations in coronavirus replication, particularly during early infection phases, and outline hypothesis regarding translation–transcription conflicts, postulating the existence of mechanisms that resolve these issues. In cells infected by coronaviruses, abundant structural proteins are synthesized from subgenomic RNA fragments (sgRNAs) produced via discontinuous transcription. During elongation, RdRP can skip large sections of the viral genome, resulting in the creation of shorter sgRNAs that reflects the stoichiometry of viral structural proteins. Although the precise mechanism of discontinuous transcription remains unknown, we discuss recent hypotheses involving long‐distance RNA–RNA interactions, helicase‐mediated RdRP backtracking, dissociation and reassociation of RdRP, and RdRP dimerization.

## Graphical abstract

The steps involved in a Coronavirus infection are interconnected and exhibit complex kinetics, with some processes depending on each other, while others occur independently. Understanding the basic kinetics of major processes like translation, transcription, and virion assembly in the Coronavirus life cycle adds temporal resolution to the classical view. Notably, the translation of the initial gRNA must be inhibited to allow for its full‐length transcription.

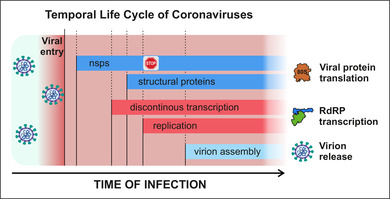

## 1 Introduction

The emergence of COVID‐19 in 2019, Middle East respiratory syndrome (MERS) in 2012, and severe acute respiratory syndrome (SARS) in 2002 posed significant global public health challenges in the 21st century. These respiratory diseases are caused by three novel coronaviruses (CoV)—SARS‐CoV‐2, MERS‐CoV, and SARS‐CoV, respectively—belonging to the Coronaviridae family (Al‐Salihi and Khalaf 2021). The first human CoVs discovered, HCoV‐229E and HCoV‐OC43, were identified in the 1960s and are known to cause respiratory conditions (Peiris and Poon 2021). This family derives its name from the crown‐like morphology observed under an electron microscope (Al‐Salihi and Khalaf 2021). CoVs are classified under the order Nidovirales and comprise two subfamilies: Orthocoronavirinae, which includes the most pathogenic viruses, and Letovirinae. The Orthocoronavirinae subfamily is divided into four genera: *Alpha*‐ (*α*), *Beta*‐ (*β*), *Gamma*‐ (*γ*), and *Deltacoronavirus* (*δ*), with a potential additional genus, *Epsilon* (*ε*) (Peiris and Poon 2021; Wang et al. 2018). MERS‐CoV and both SARS‐CoV and SARS‐CoV‐2 belong to the *Betacoronavirus* genus.

CoVs can infect both avian and mammalian species, exhibiting extensive genetic diversity in humans and reservoir species, particularly bats. With a wide range of strains, variants, and lineages across species, these viruses can cause severe respiratory illnesses and pose a significant life‐threatening risk (Coronaviridae Study Group of the International Committee on Taxonomy of Viruses 2020). Immunocompromised individuals, the elderly, and those with diabetes are at a higher risk of severe COVID‐19, as they tend to experience organ dysfunction, more severe pneumonia, and prolonged viral shedding (Wünsch et al. 2022). These high‐risk groups are also more susceptible to long COVID (also referred to as “post‐acute sequelae of COVID‐19”), which affects at least 10% of SARS‐CoV‐2‐infected individuals. Long COVID is characterized by prolonged recovery, and involves over 200 symptoms across multiple organ systems, with an estimated 65 million global cases (Davis et al. 2023; Wünsch et al. 2022).

A deep understanding of CoV biology and structure provides a framework for developing diagnostic and treatment strategies, as demonstrated by several successful approaches: RT‐qPCR tests, rapid antibody‐based assays, monoclonal antibodies, and replication inhibitors such as remdesivir and nirmatrelvir.

In this work, we review the current knowledge of the CoV life cycle and protein components, with a particular focus on the transcription machinery and its kinetics. We provide new insights into key stages of the CoV life cycle with a temporal perspective, raising questions that could lead to new regulatory and therapeutic strategies. As CoVs are positive‐sense single‐stranded RNA (ssRNA+), some of these insights apply to all ssRNA(+) viruses.

## 2 Virus Morphology and Genomic Architecture

**FIGURE 1:**
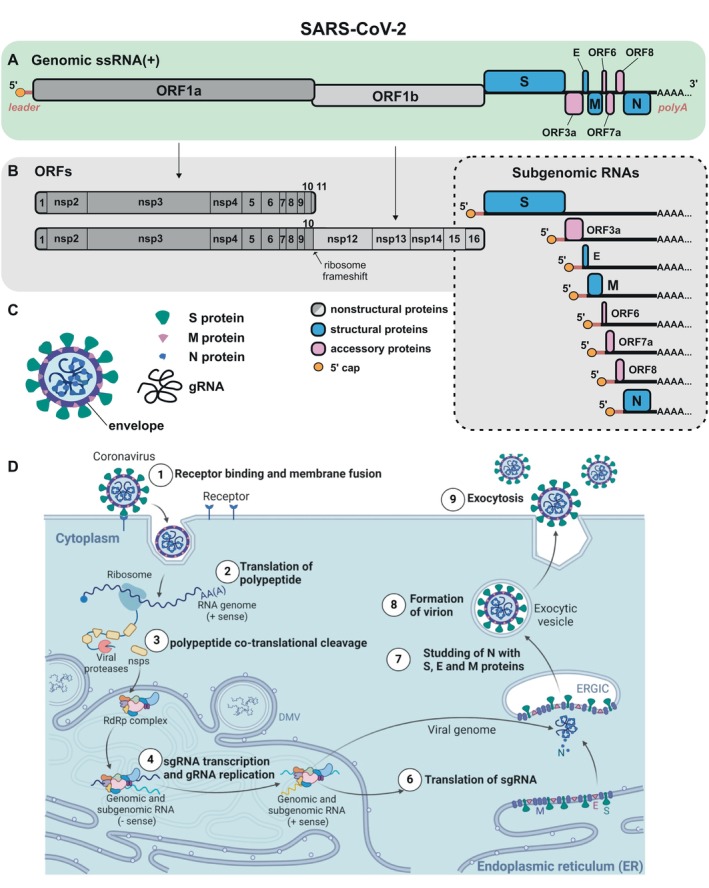
Basic features of coronaviruses (CoVs) (on the basis of Bresson et al. 2020; Hartenian et al. 2020; Hulo et al. 2011). (A) Architecture of CoV genomic ssRNA(+). The gRNA has a 5′ cap and a 3′ polyadenylated tail. The genome is organized to encode essential nonstructural proteins (ORF1a and ORF1b) and structural proteins (spike, S; envelope, E; membrane, M; and nucleocapsid, N) necessary for the formation of new virions. (B) Open reading frames in the CoV genome. The translation of ORF1b is allowed by ribosomal frameshifting. Subgenomic RNAs (sgRNAs) are generated by a discontinuous transcription mechanism. (C) Structure of mature CoV particles. Virions are spherical, enveloped with a round surface that forms corona‐like spikes around them. (D) The life cycle of CoVs. Key steps include attachment, entry, translation, replication, assembly, and the release of new virions. This cycle begins when the viral spike (S) protein binds to a specific receptor on the host cell surface. After binding, the virus enters the host cell, typically through a fusion of the viral envelope with the cell membrane or endosomal membrane. Inside the cell, the viral genome is released and translated to produce viral proteins necessary for replication and the production of new virions. The assembled virions are then transported to the cell surface and released, ready to infect new cells.

CoVs share common structural features and are recognized as the largest positive‐strand RNA viruses, with genome sizes of approximately 30 kilobases (kb) and virion diameters around 125 nm (Steiner et al. 2024) (Figure 1). The genomic RNA (gRNA) has a 5′ cap and a 3′ polyadenylated tail (Figure 1A). More than half of the viral genome at the 5′ end encodes open reading frames (ORF1a and ORF1b), which are directly translated from the gRNA to produce two polyproteins. The polyproteins undergo cotranslational proteolysis into 16 nonstructural proteins (nsps) (Figure 1B). Nsps are available from the onset of viral infection as their translation does not depend on viral transcriptional activity. These proteins are essential for viral RNA synthesis and facilitating interactions with the host cell during infection. Table 1 highlights the functions of all nonstructural and structural viral proteins during CoV infection.

**TABLE 1:** Characteristics of coronavirus nonstructural and structural proteins (on the basis of (Malone et al. 2022; Steiner et al. 2024; Wang, Grunewald, and Perlman 2020; Yan et al. 2022).

Protein name	aa length for SARS‐CoV‐2^a^	Functions	Reference
Nonstructural proteins (nsps)			
nsp1	180	‐ Binds to ribosomes to block mRNA entry, causing translational halt and degradation of host mRNAs lacking the 5′ viral leader sequence. ‐ Hinders the induction and interferon signaling. ‐ Interferes with nuclear mRNA export by interacting with NXF1 (nuclear RNA export factor 1) and NXT1 (nuclear transport factor 2‐like export factor 1).	Ghaleh et al. (2023), Schubert et al. (2020).
nsp2	638	‐ Binds to host proteins (PHB1, PHB2, WASH), possibly disrupting host signaling. ‐ Interacts with translational repressors GIGYF2 and EIF4E2, hindering IFNβ expression. ‐ Weakens the suppression ability of the cap‐binding eIF4E‐homologous protein (4EHP)‐GIGYF2 complex.	Cornillez‐Ty et al. (2009); Xu et al. (2022); Zou et al. (2022).
nsp3	1945	‐ Process polyyprotein via papain‐like protease (PLpro) activity. ‐ Interacts with the nucleocapsid (N) protein using ubiquitin‐like1 (Ubl1) and acidic domains. ‐ Macrodomains (Mac1, Mac2, Mac3) exhibit mono‐ADP‐ribosylhydrolase activity. ‐ Contributes in forming double‐membrane vesicles (DMVs), inducing membrane curvature, constituent of the DMV pore.	Alhammad et al. (2021); Claverie (2020); Klemm et al. (2020); Qin et al. (2023); Shi, Feng, and Zhang (2021); Shin et al. (2020).
nsp4	500	‐ Participate in formation of replication organelles by altering cell membranes, creating pores for RNA transport, and interacting with host factors to facilitate viral replication.	Angelini et al. (2013); Lin et al. (2023); Oudshoorn et al. (2017).
nsp5	306	‐ Called the main protease (Mpro) or Picornavirus 3C‐like protease, cleavage of viral polyproteins. ‐ Inhibits NF‐κB activation by processing NLRP12 and TAB1, also cleaves RIG‐I and IRF3. ‐ Enhances viral replication by cleaving the host factor RNF20. ‐ Interferes with host sensors, promoting MAVS degradation. ‐ Sequesters G3BP1, disrupting stress granule formation.	Anand et al. (2003); Liu et al. (2021); Moustaqil et al. (2021); Zhang, Wang, and Cheng (2021); Zheng et al. (2022); Zhu et al. (2017).
nsp6	290	‐ Potential transmembrane scaffold protein. Acts as a coordinator of DMV clusters and selectively replenishes them with lipids from lipid droplets, playing a role in the formation of the replication organelle and linking it to the ER. ‐ Inhibits the phosphorylation of STAT1 and STAT2 hindering interferon signaling. ‐ Interacts with TBK1, leading to interference with host sensors.	Angelini et al. (2013); Oostra et al. (2008); Ricciardi et al. (2022); Sun et al. (2022); Xia et al. (2020).
nsp7	83	‐ Function as nsp12 RdRP cofactor together with nsp8. Mediate RdRP activity as holoenzyme subunit. ‐ Hinders IFNα signaling.	Kirchdoerfer and Ward (2019); Xia et al. (2020); Zhai et al. (2005).
nsp8	198	‐ Functions as cofactors for nsp12 RdRP alongside nsp7, by serving as a subunit of the RdRP holoenzyme. ‐ Was suggested as potential 3′‐terminal adenylyltransferase and primase in the past. ‐ Interferes with protein trafficking by binding to the signal recognition particle complex.	Banerjee et al. (2020); Imbert et al. (2006); Kirchdoerfer and Ward (2019); Tvarogová et al. (2019); Zhai et al. (2005).
nsp9	113	‐ RNA‐binding protein, disrupts protein trafficking by binding to the signal recognition particle complex. ‐ Putative NiRAN UMP‐transferase substrate in RNA capping, also serving as an adaptor for nsp14 and nsp16. ‐ Involved in priming RNA synthesis as an NMPylation target for the NiRAN domain.	Banerjee et al. (2020); Egloff et al. (2004); Slanina et al. (2021); Wang, Svetlov, and Artsimovitch (2021); Yan et al. (2022).
nsp10	139	‐ Serves as a cofactor for nsp14 and nsp16 by forming a heterodimer, enhancing the activity of 2‐O‐MT (nsp16 methyltransferase) and ExoN (nsp14 exonuclease). ‐ Regulates ribosomal frameshifting.	Bhatt et al. (2021); Bouvet et al. (2010); Decroly et al. (2011); Lin et al. (2021); Ma et al. (2015); Smith et al. (2015).
nsp11	13	‐ Short peptide.	Rogstam et al. (2020)
nsp12	932	‐ Catalytic subunit of the RdRP. Core element together with nsp7 and two subunits of nsp8. ‐ Involved in initiating RNA synthesis, its NiRAN domain performs NMPylation on nsp9. ‐ Suggested role in viral RNA capping by functioning as GDP‐PRNTase, transferring RNA to GDP/GTP after NiRAN RNAylates nsp9.	Kirchdoerfer and Ward (2019); Park et al. (2022); Slanina et al. (2021); Wang, Svetlov, and Artsimovitch (2021); Xu et al. (2003); Yan et al. (2022).
nsp13	601	‐ Helicase activity at the zinc‐binding domain. ‐ RNA 5′‐triphosphatase activity, crucial for viral RNA capping. ‐ Facilitates backtracking during RNA synthesis. ‐ Inhibits phosphorylation of IRF3, TBK1, STAT1, and STAT2, disrupting interferon signaling and host sensors.	Chen et al. (2022); Fung et al. (2022); Ivanov et al. (2004); Ivanov and Ziebuhr (2004); Lei et al. (2020); Malone et al. (2021); Yuen et al. (2020).
nsp14	527	‐ 3′ to 5′ exoribonuclease (ExoN) activity responsible for nascent RNA proofreading during viral RNA replication. ‐ N7‐MTase activity. ‐ Plays a role in mRNA capping (5′ cap) during viral RNA replication. ‐ Inhibits host translation with the assistance of nsp10. ‐ Disrupts IFN‐induced antiviral activity by targeting IFNAR1, playing role in lysosomal degradation and preventing nuclear localization of IRF3.	Bouvet et al. (2010); Chen et al. (2009); Eckerle et al. (2007, 2010); Hsu et al. (2021); Lei et al. (2020).
nsp15	346	‐ Endoribonuclease (EndoU) with uridine specificity. ‐ Plays a role in reducing content of pathogen‐associated molecular patterns (PAMPs) by cleaving 5′‐polyuridine tracts from viral RNA negative strands. ‐ Prevents IRF3 from moving to the nucleus, disrupting interferon signaling.	Bhardwaj et al. (2006); Frazier et al. (2022); Ivanov et al. (2004); Kindler et al. (2017); Yuen et al. (2020).
nsp16	298	‐ Mg2+ and nsp10‐dependent 2′‐O‐methyltransferase, playing a role in the capping of viral RNA. ‐ Suppresses splicing by binding to recognition domains for mRNA of U1 and U2 (spliceosome RNA components). ‐ Protects viral RNA from being recognized by MDA5.	Banerjee et al. (2020); Decroly et al. (2008); Park et al. (2022); Walker et al. (2021).
Structural proteins			
Spike (S)	1273	‐ The S1 subunit has the receptor‐binding domain (RBD) that allows the virus to bind to the host receptor (ACE2 for SARS‐CoV‐2). ‐ The S2 subunit is responsible for membrane fusion between the virus and host cell. ‐ Undergoes proteolytic cleavage by host proteases like furin, separating S1 and S2 subunits to prepare for viral fusion. ‐ Induces IRF3 degradation, interfering with host sensors. ‐ Prevents JAK1 and STAT1 interaction, blocking interferon signaling.	Freitas, Crum, and Parvatiyar (2021); Taha et al. (2023); Zhang et al. (2021).
Envelope (E)	75	‐ Involved in viral assembly in conjunction with M. ‐ Exhibits viroporin function, forming calcium‐selective ion channels that disrupt host pathogenesis activity.	Boson et al. (2021); Bracquemond and Muriaux (2021); Mandala et al. (2020).
Membrane (M)	222	‐ Plays a central role in virion assembly, interacting with N protein for nucleocapsid incorporation and E protein for regulating S protein intracellular trafficking, influencing viral envelope shape. ‐ Interferes with IFN‐I signaling by degrading TBK1, inhibiting STAT1 phosphorylation, and interacting with IKKϵ, TRAF3, and MDA5. ‐ Impairs MAVS activation and blocks IRF3 nuclear translocation.	Boson et al. (2021); Fu et al. (2021); Lu et al. (2021); Mahtarin et al. (2022); Sui et al. (2021); Thomas (2020); Xia et al. (2020); Zhang et al. (2021).
Nucleocapsid (N)	419	‐ Consist of two functional domains: the N‐terminal domain (NTD) which binds to RNA and the C‐terminal domain (CTD) that facilitates protein oligomerization. These regions work together to condense and package the viral genome. ‐ Facilitates genome packaging and virion assembly by binding to the viral RNA genome and incorporating it into a helical ribonucleoprotein structure within the viral particle, creating a nucleocapsid. ‐ Counteracting antiviral RNAi responses, and nuclear translocation of STAT1 and STAT2, aiding the virus in evading host immune defenses. ‐ Disrupts stress granule formation by sequestering G3BP1, hindering RIG‐I activation, and preventing IRF3 and MAVS activation.	Cubuk et al. (2021); Gao et al. (2021); Gori Savellini et al. (2021); Lu et al. (2021); Luo et al. (2021); Morse et al. (2023); Mu et al. (2020); Mu et al. (2020); Oh and Shin (2021); Wang et al. (2021); Zheng et al. (2022).

Abbreviations: DMV, double‐membrane vesicle; EIF4E2, eukaryotic translation initiation factor 4E family member 2;G3BP1, Ras GTPase‐activating protein‐binding protein 1;GDP, guanosine diphosphate; GIGYF2, GRB10‐interacting GYF protein 2; GTP, guanosine triphosphate; IFNAR1, interferon alpha and beta receptor subunit 1; IKKϵ, Inhibitor of nuclear factor kappa‐B kinase subunit epsilon; IRF, interferon regulatory factor; IRN, interferon; JAK1, Janus kinase 1; MAVS, mitochondrial antiviral signaling protein; MDA5, melanoma differentiation‐associated protein 5; Mpro, main protease; N7‐MTase (guanine‐N7‐)‐methyltransferas; NiRAN, nidovirus RdRP‐associated nucleotidyltransferase; NLRP, NLR family pyrin domain‐containing protein; NMP, nucleotidyl monophosphate; NXF1, nuclear RNA export factor 1; NXT1, nuclear transport factor 2‐like export factor 1; PAMP, pathogen‐associated molecular pattern; PLpro, papain‐like protease; PRNT, polyribonucleotidyltransferase; RdRP, RNA‐dependent RNA polymerase; RIG‐I, retinoic acid‐inducible gene I; RNF20, ring finger protein 20; SARS‐CoV‐2, severe acute respiratory syndrome coronavirus 2; STAT, signal transducer and activator of transcription; TAB1, transforming growth factor‐β activated kinase 1 (TAK1)‐binding protein 1; TBK1, TANK‐binding kinase 1; TRAF3, tumor necrosis factor (TNF) receptor‐associated factor 3, Ubl, ubiquitin‐like; UMP, uridine monophosphate.

^a^ Wuhan‐Hu‐1 isolate (GenBank entry MN908947.3).

**TABLE 2:** Selected nucleoside analog inhibitors of SARS‐CoV‐2 RdRP (on the basis of Xu and Zhang 2023).

Mechanism of inhibition	Compound	Chemical formula	Type of modification	Reference
Immediate chain termination	AT‐9010	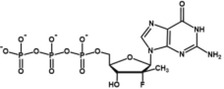	2′ ribose modification	Shannon et al. (2022)
	Sofosbuvir	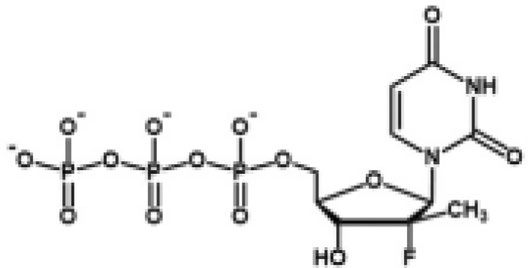	2′ ribose modification	Jockusch et al. (2020)
	Cordycepin	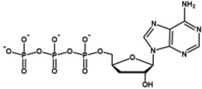	3′ ribose modification	Rabie (2022)
	Stavudine	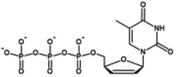	2′, 3′ ribose modification	Jockusch et al. (2020)
	Carbovir	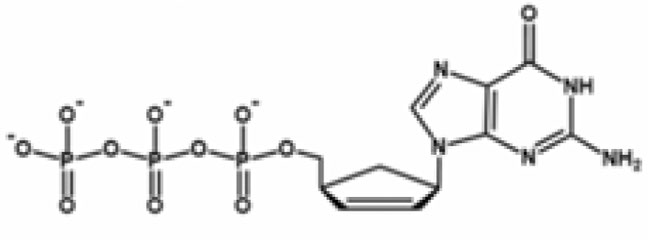	2′, 3′ ribose modification	Jockusch et al. (2020)
	Ganciclovir	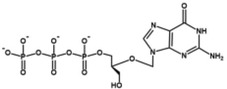	Acyclic modification	Jockusch et al. (2020)
Delayed chain termination	Remdesivir	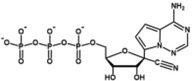	1′ ribose modification	Bravo et al. (2021)
	Cidofovir	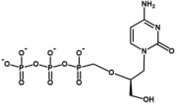	Acyclic modification	Jockusch et al. (2020)
Mutagenesis	Molnupiravir	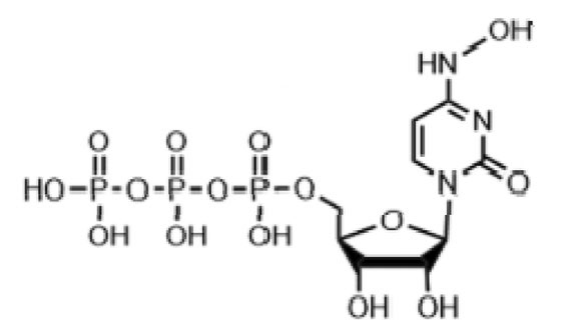	Cytidine modification	Kabinger et al. (2021)
	Favipiravir	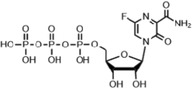	Purine modification	Shannon et al. (2020)

The remaining 3′ portion of the gRNA encodes four structural proteins, as well as genes encoding accessory proteins (Zhou et al. 2020; Zhu et al. 2020). In contrast to ORF1a and 1b, structural and accessory proteins are produced through subgenomic messenger RNAs (sg‐mRNAs or sgRNA(+)), arranged in nested sets. This mechanism allows the simultaneous production of template sgRNA(−) by RdRP and translation of ORF1a and ORF1b by ribosomes, both crucial for infection kinetics. The structural proteins of SARS‐CoV‐2 include the envelope (E), membrane (M), nucleocapsid (N), and spike (S) proteins. The spike protein plays a critical role in viral entry, while the other proteins coordinate various steps in viral replication, including the assembly, packaging, and budding of new virions. Accessory proteins, though not essential for viral replication in cell culture, serve additional functions (Jungreis, Sealfon, and Kellis 2021) (Figure 1C,D)

## 3 Coronavirus Life Cycle

### 3.1 Attachment and Entry

The initial binding of the viral particle to a host cell is triggered by the interaction between the spike (S) protein and its corresponding receptor. This mechanism is key in determining the range of host species and the specific tissues targeted by CoVs. The S protein consists of two subunits: the variable S1 subunit, which facilitates receptor binding, and the conserved S2 subunit, which undergoes conformational changes leading to the fusion of the viral and cellular membranes (Wang, Grunewald, and Perlman 2020).

The interaction of the S protein with the host cell receptor is essential for infection and cross‐species transmission, making it the primary target for neutralizing antibodies. In many CoVs, the S protein is cleaved during release from infected cells, often mediated by a furin‐like protease (Millet and Whittaker 2014). This cleavage separates the RBD from the fusion domains of the S protein (Belouzard, Chu, and Whittaker 2009). After receptor binding, the virus typically requires entry into the host cell cytosol. This is often achieved through a secondary proteolytic cleavage of the S protein by transmembrane serine protease 2 (TMPRSS2) or cathepsins (Kleine‐Weber et al. 2018; Park et al. 2016). After cleavage at the S2 site, a fusion peptide is exposed, enabling the formation of an antiparallel six‐helix bundle from two heptad repeats in the S2 region (Bosch et al. 2003). The assembly of this bundle facilitates membrane fusion and the subsequent release of the viral genome into the cytoplasm. Fusion typically occurs at the plasma membrane, though in some cases, it can happen within acidified endosomes (White and Whittaker 2016).

Sidebar 1: The S Protein Determines the Type of Host Cell

The location of the receptor‐binding domain (RBD) within the S1 region varies across different CoVs. For example, in some cases like murine hepatitis virus (MHV), the RBD is located at the N terminal of S1 (Wang, Grunewald, and Perlman 2020). In contrast, viruses such as SARS‐CoV (Wong et al. 2004), MERS‐CoV (Wang et al. 2013), HCoV‐229E (Bonavia et al. 2003), HCoV‐HKU1 (Qian et al. 2015), HCoV‐NL63 (H.‐X. Lin et al. 2008), and TGEV (Godet et al. 1994) have the RBD at the C terminal of S1.

Many δ‐CoVs and α‐CoVs use aminopeptidase N (APN) as their cellular receptor (Li et al. 2018; Wang et al. 2018). APN, also known as CD13, is a heavily glycosylated homodimeric protein and a zinc‐binding cell‐surface protease predominantly found in respiratory, enteric, and neural tissues. On the other hand, β‐CoVs like SARS‐CoV and SARS‐CoV‐2 use angiotensin‐converting enzyme 2 (ACE2) as their receptor for cell entry (Li et al. 2003). ACE2 is primarily expressed in lung and small intestine epithelial cells, which are the main targets for SARS‐CoV, SARS‐CoV‐2, and HCoV‐NL63 (Hofmann et al. 2005). ACE2 is also present in other tissues, such as the heart and kidneys (Hamming et al. 2004). ACE2 functions as a zinc‐binding cell‐surface carboxypeptidase, playing a crucial role in regulating cardiac function and blood pressure. β‐CoVs, like MERS‐CoV, use dipeptidyl‐peptidase 4 (DPP4) as their cellular receptor (Raj et al. 2013). DPP4, also known as CD26, is a membrane‐bound exoprotease distributed across various tissues, involved in the cleavage of dipeptides from chemokines, cytokines, and hormones. Critical residues for binding to the RBD of both human and camel DPP4 are highly conserved, facilitating the zoonotic transmission of MERS‐CoV (Li 2015).

### 3.2 Translation of Viral Proteins

The next phase in the CoV life cycle involves the translation of two large polyproteins, pp1a and pp1b, from the viral genomic RNA (gRNA). To translate the second polyprotein, from ORF1ab, the virus employs a slippery sequence (5′‐UUUAAAC‐3′) followed by an RNA pseudoknot, which induce ribosomal frameshifting from the ORF1a reading frame to ORF1b (Wang, Grunewald, and Perlman 2020). Typically, the ribosome unravels the pseudoknot structure and continues translating until it encounters the stop codon of ORF1a. However, the pseudoknot can slow ribosome elongation, causing the ribosome to pause at the slippery sequence. This pause triggers a change in the reading frame, enabling the ribosome to shift back by one nucleotide (−1 frameshift) before resuming translation into ORF1b (Baranov et al. 2005; Brierley, Digard, and Inglis 1989).

Various in vitro studies have estimated the incidence of ribosomal frameshifting; however, its efficiency during actual virus infection in vivo has not been definitively determined. Some studies suggest that frameshifting efficiency in infected cells may vary, ranging from 10% to 15% or as high as 15% to 30%, depending on cellular factors (Bhatt et al. 2021; Kelly et al. 2020; Kelly, Woodside, and Dinman 2021; Plant et al. 2005). The frameshifting mechanism is believed to regulate the ratio between ORF1a and ORF1b proteins, potentially delaying ORF1b production until ORF1a creates an environment suitable for RNA replication (Araki et al. 2010).

Moreover, the RNA pseudoknot must first be unfolded by the translating ribosome and then refolded to induce frameshifting in subsequent ribosome. This means that the spacing between ribosomes affects the frameshifting rate: larger spacing increases the chances of pseudoknot refolding and frameshifting. Since translation elongation is fairly constant, ribosome spacing is set during translation initiation. Thus, we propose that the probability of ribosome frameshifting is directly linked to the translation initiation rate.

The nonstructural proteins (nsps) 1–11 and 1–16 are encoded within the polyproteins pp1a and pp1ab, respectively. CoVs typically exhibit two types of polyprotein cleavage activities. The primary cleavage is carried out by a single papain‐like proteases (PLpro) in nsp3, which cleaves nsp1, nsp2, and nsp3. Some viruses, such as arteriviruses (also belonging to the Nidovirales order), encode two PLpros with distinct cleavage sites. Additionally, many viral PLpro enzymes possess deubiquitinase activity, which helps counteract certain host antiviral defense mechanisms by removing ubiquitin and ISG15 conjugates, thereby modulating immune responses (Mielech et al. 2014). The remaining 11 cleavage events are mediated by the main protease (Mpro), also known as nsp5 (Anand et al. 2002; Stobart et al. 2012). Mpro is frequently referred to as the 3C‐like protease (3CLpro) due to its distant relation to the 3C proteases of picornaviruses. Both PLpro and Mpro are prime targets for antiviral drug development due to their critical roles in the viral life cycle (Mielech et al. 2014; Yang et al. 2005).

### 3.3 Transcription and Replication of Viral RNAs

Coronaviruses employ a distinctive transcription method, producing genomic RNA (gRNA) and subgenomic RNAs (sgRNAs), with the sgRNAs serving exclusively as mRNAs (Wang, Grunewald, and Perlman 2020). All positive‐sense sgRNAs(+) share a 3′ coterminal arrangement with the full‐length viral genome, creating a set of nested RNAs, a key feature of the Nidovirales order. Both gRNAs and sgRNAs are synthesized via negative‐strand (RNA(−)) intermediates, which are about 1% as abundant as their positive‐sense counterparts, containing both antileader and polyuridylate sequences (Sethna, Hofmann, and Brian 1991). Therefore, gRNA replication involves two rounds of transcription: ssRNA(+) to ssRNA(−) and ssRNA(−) to ssRNA(+).

**FIGURE 2:**
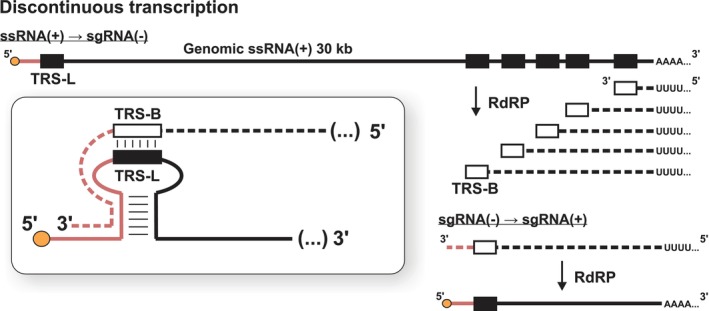
Discontinuous transcription of subgenomic RNAs (sgRNA) (on the basis of Ge et al. 2024; V'kovski et al. 2021).

The synthesis of sgRNAs involves a unique discontinuous transcription process. The 6‐ to 9‐nt‐long transcription regulatory sequences (TRS) are crucial in this process, facilitating RNA–RNA joining during transcription (Sawicki, Sawicki, and Siddell 2007). Body TRS (TRS‐B) base pairs with the TRS‐L present in the leader sequence at the 5′ of the ssRNA(+) (Fung and Liu 2019; Zúñiga et al. 2004) (Figure 2). The RNA‐dependent RNA polymerase (RdRP) may pause at the TRS‐B sequences, switching between amplification of the leader sequence at the 5′ end of the genome or elongation to the next TRS‐B.

In SARS‐CoV‐2, all structural proteins, including the nucleocapsid (N), matrix (M), envelope (E), and spike (S) proteins, are encoded at the 3′ end of the genome and translated from sgRNAs (Kim et al. 2020). Discontinuous transcription leads to the synthesis of sgRNA(−), followed by high‐copy sgRNA(+) mRNAs synthesis (Enjuanes et al. 2006; Fung and Liu 2019; Sawicki and Sawicki 1998). The 5′ leader sequence is necessary for ribosome binding and translation initiation, while the 3′ end varies in length (Nakagawa, Lokugamage, and Makino 2016).

Sidebar 2: The Evolution of Coronaviruses Is Facilitated by a Template‐Switching Mechanism

Coronaviruses also exhibit the ability to undergo recombination through both homologous and nonhomologous mechanisms, primarily due to the strand‐switching capabilities of RdRP. This recombination significantly contributes to viral evolution and is the basis for reverse genetics techniques, such as engineering viral recombinants (Kuo et al. 2000; Lai et al. 1985).

Through homologous recombination, the virus can exchange genetic material between different strains or even different species, which can result in the emergence of novel viral variants with enhanced pathogenicity, immune evasion capabilities, or altered host range. Nonhomologous recombination, on the other hand, can lead to more drastic genetic rearrangements, including deletions, insertions, or duplications of genetic sequences, further accelerating viral diversity.

The strand‐switching mechanism of the RdRP is central to this recombination process. During replication, the RdRP can dissociate from the template RNA strand and reassociate with a different RNA strand, effectively combining genetic material from separate RNA molecules allowing CoVs to mix and match genetic sequences. The understanding of this mechanism has facilitated the development of reverse genetics systems. These systems allow scientists to manipulate the viral genome in the laboratory, creating recombinant viruses with specific genetic modifications.

The generation of sgRNA involves an unusual process where the RdRP skips over a large section of the viral genome. This leads to the production of short sgRNA(−), which are then transcribed to produce sgRNA(+), mRNA for the virus's structural proteins (Sola et al. 2011). Discontinuous transcription is controlled by transcription regulatory sequences (TRS), specifically TRS‐B and TRS‐L. The TRS‐L sequence is situated in the stem‐loop of the 5′ leader sequence, allowing it to base pair.

### 3.4 Replication Organelles, Assembly of Virions, and Release

**FIGURE 3:**
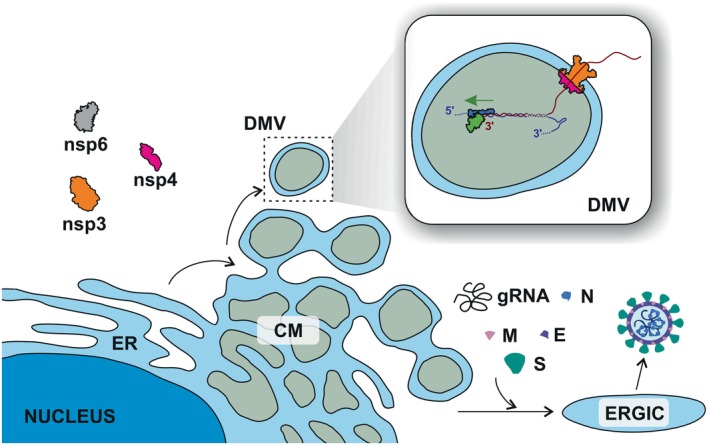
Formation of replication organelles. Convoluted membranes (CM) serve as an intermediate structure, from which double‐membrane vesicles (DMVs) are generated (Knoops et al. 2008). The organelle formation involves three viral proteins: nsp3, nsp4, and nsp6 (Ricciardi et al. 2022; Zimmermann et al. 2023) Nsp3 and nsp4 are components of molecular pores that traverse the DMV membranes. These DMV pores enable the transport of nascent viral RNA, synthesized by RdRP (right panel), into the cytoplasm (Huang et al. 2024). The assembly of virions takes place in the endoplasmic reticulum–Golgi intermediate compartment (ERGIC) (de Haan and Rottier 2005).

The replication of CoVs, like other positive‐sense RNA viruses, takes place in the cytoplasm of host cells. CoV infection induces the formation of various small organelles, which create compartments within the cell. These organelles are thought to play different roles in the virus life cycle. The following structures have been described: double‐membrane vesicles (DMVs), convoluted membranes (CMs), double‐membrane spherules (DMSs), large virion‐containing vacuoles (LVCVs), tubular bodies (TBs), and cubic membrane structures (CMSs) (Ulasli et al. 2010). LVCVs likely function as secretory organelles for virions assembled in the endoplasmic reticulum–Golgi intermediate compartment (ERGIC) (Snijder et al. 2020; Ulasli et al. 2010). The roles of other virus‐induced organelles remain speculative, but the most established role is for DMVs, connected to CMs, which appear to serve as transcription sites for viral RNA (Figure 3).

The encapsulation of replication machinery and newly synthesized viral RNA within these replication organelles helps the virus evade cellular antiviral mechanisms, particularly those that detect double‐stranded RNA (dsRNA). The presence of DMVs in cells was observed as early as 2 h postinfection (hpi) with SARS‐CoV and MHV (Knoops et al. 2008; Ulasli et al. 2010). However, in the case of SARS‐CoV‐2 infection, DMV formation occurs around six hpi (Cortese et al. 2020; Eymieux et al. 2021). Viral proteins such as nsp8, nsp3, nsp4, and the nucleocapsid (N) protein, along with viral RNA, were shown to colocalize with DMVs (Ulasli et al. 2010). Both viral ssRNA and dsRNA were found inside DMVs (Andronov et al. 2024; Snijder et al. 2020).

The replication organelles originate from host cell membranes, but viral proteins are essential for their formation. Nsp6 plays a key role in the restructuring of the endoplasmic reticulum (ER) to form DMVs and in linking DMVs to the ER and lipid droplets, which are associated with the growth of DMVs (Ricciardi et al. 2022). DMVs contain molecular pores made of viral proteins nsp3 and nsp4 (Figure 3) (Wolff et al. 2020). These pores are hexameric, crown‐shaped structures that span the DMV membranes. A channel in the center of the oligomer structure is wide enough to allow the passage of a single‐stranded RNA (Huang et al. 2024). The discovery of these molecular pores supports the role of DMVs as replication sites for viral RNA. It is likely that newly synthesized RNA is exported from DMVs through these pores into the cytoplasm, where it undergoes translation or is packaged into virions, however, exact mechanism of this process remains unknown.

The precise cellular localization of viral transcription during the initial stages of infection remains to be elucidated. If the viral gRNA can be replicated solely within DMVs, the RNA strand released from the infecting virion would have to undergo multiple rounds of translation to produce the requisite amount of proteins for the formation of replication organelles. It can be postulated that transcription initially occurs in the cytoplasm, and that the transfer of this process into DMVs only enhances its efficiency in later stages of infection, however, more definitive answer require further research.

The assembly of a functional virion requires newly synthesized ssRNA(+) and the structural proteins (S, E, M, and N), which are translated from sgRNAs and incorporated into the ER. These structural proteins then follow the secretory pathway toward the ERGIC (Wang, Grunewald, and Perlman 2020). Inside the ERGIC, the viral gRNA is encapsulated by the N protein and buds into ERGIC membranes containing the structural proteins, leading to the formation of mature virions (de Haan and Rottier 2005).

Once assembled, virions are transported to the cell surface in vesicles and released via exocytosis. It remains unclear whether the virions hijack a conventional Golgi pathway for large cargo transport or whether the virus follows a unique pathway for exit. A host protein called valosin‐containing protein (VCP/p97) was identified through genome‐wide screening as being required for the release of CoVs from endosomes (Wong et al. 2015).

In many CoVs, S proteins that are not incorporated into virions are transported to the cell surface. At the surface, the S protein can facilitate cell–cell fusion between infected cells and neighboring uninfected cells. This fusion forms multinucleated cells, or syncytia, allowing the virus to spread within an infected host without being detected or neutralized by virus‐specific antibodies (Wang, Grunewald, and Perlman 2020). This pathway of cell infection would exhibit markedly different dynamics. Initially, both gRNA and RdRP would not be limiting factors at the beginning of infection. However, a newly infected cell would still possess a fully operational innate immunity and an unaffected gene expression system. Consequently, predicting the kinetics of viral infection during cell–cell fusion is challenging.

## 4 Transcription and Replication Machinery

The large size of CoV genomes require efficient replication mechanisms to enable fast and accurate duplication of genetic material. Current progress has highlighted that, in addition to the core machinery, various viral and host factors are involved in this process (reviewed in Steiner et al. 2024). Although the assembly of RdRP and its interaction with additional factors are still not fully understood, considerable information about its structure and functionality is available (reviewed in Malone et al. 2022).

### 4.1 RdRP Structure

**FIGURE 4:**
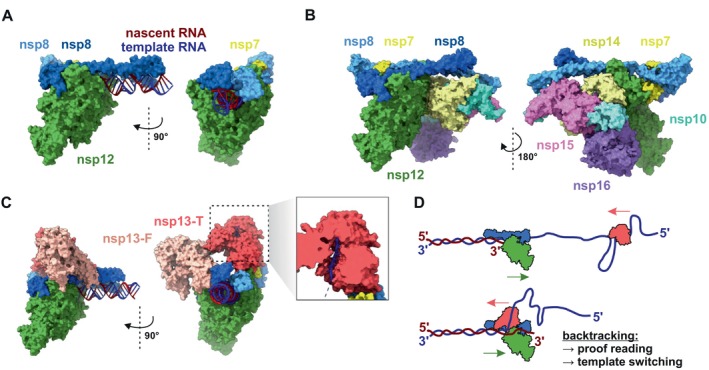
Transcription and replication machinery. (A) The structure of replicating RNA‐dependent RNA polymerase (RdRP) with core subunits (nsp12, nsp7, nsp8) and dsRNA intermediate is shown (PDB: 7DTE) (Wu et al. 2021). Two nsp8 subunits form a scaffold that supports the template–product RNA hybrid. (B) Model of RdRP along with the predicted positions of its auxiliary proteins: nsp14, nsp15, nsp16, and nsp10 (Perry et al. 2021). (C) The RdRP structure with two subunits of the nsp13 helicase. The nsp13‐T subunit is positioned on the “thumb” side of nsp12, while the nsp13‐F subunit is situated on the “finger” side of nsp12. A close‐up view reveals the template RNA strand passing through nsp13‐T during polymerase backtracking (PDB: 7RDY) (Chen et al. 2022). (D) Directionality of RdRP and nsp13 helicase. The RdRP moves along the template strand in a 3′→5′ direction, whereas nsp13 helicase unwinds dsRNA in the 5′→3′ direction. This indicates that nsp13 primarily functions in RdRP backtracking, which is crucial for maintaining RdRP fidelity, and was suggested to play a role in discontinuous transcription.

Structural studies of the SARS‐CoV‐2 RdRP complex, bound to the dsRNA template–product helix, have unveiled the structural architecture and intricate mechanism of viral RNA synthesis. The replicating core of the enzyme comprises one unit of nsp12, two units of nsp8, and one nsp7 protein (Figure 4A). The primary polymerase activity is attributed to the nsp12 “right hand” structure, featuring fingers, palm, and thumb domains. Each nsp8 subunit bound to nsp12 possesses a long N‐terminal, positively charged α‐helix structure protruding from the polymerase complex. Together, these proteins create a structure that nonspecifically binds, via phosphate backbone, the template–product dsRNA, potentially contributing to high polymerase processivity (Campagnola et al. 2022; Hillen et al. 2020; Malone et al. 2023).

The extended replication–transcription complex may also incorporate other nonstructural viral proteins, supporting polymerase activity, including nsp9, nsp10, nsp13, nsp14, nsp15, and nsp16. While the complete structure of this extended complex remains unsolved, molecular modeling studies propose a detailed configuration based on available structural data (Figure 4B). In this model, it is suggested that the nsp15 subunit binds in proximity to the 5′ end of the template strand. The dsRNA complex navigates through nsp14 and nsp16, both assisted by their shared cofactor, nsp10. The zinc fingers found in these proteins play a crucial role in separating the two RNA strands. The template strand is then oriented away from the complex, initiating a capping process in which, among other components, nsp14 and nsp16 actively participate (Perry et al. 2021).

### 4.2 RdRP Activity

The nsp12 subunit, housing the main enzymatic activity of RdRP, contains structural motifs highly conserved among single‐stranded plus RNA viruses (Campagnola et al. 2022). It exhibits a substantially higher transcription rate than any other viral RNA polymerase (Shannon et al. 2020). This exceptional RNA polymerization velocity results from amino acid substitutions in the catalytic core compared to other RNA(+) viruses (Campagnola et al. 2022). The replacement of Glu547 with Ala in the nsp12 motif F may facilitate structural flexibility and accelerate the loading of incoming nucleotides. The potential reduction in enzyme fidelity is offset by the alteration of Gly759 to Ser in the nsp12 motif C. The serine side chain forms a hydrogen bond with the 2′ hydroxyl of the last incorporated nucleotide, positioning the ribose in an optimal orientation for the attachment of the incoming nucleotide.

Structural data shed light on the nucleotide recognition and binding process by nsp12 at the atomic level. The incoming nucleotide is bound within the active site created by the palm domain of nsp12, alongside the template RNA. The base‐pairing event triggers the closure of the active site, facilitating the incorporation of two metal ions crucial for establishing the phosphodiester bond in the synthesized strand. Following this reaction, the polymerase translocates along the template RNA, leading to the opening of the active site and creating room for the subsequent incoming nucleotide (Malone et al. 2023).

Replication of the large, nearly 30‐kb‐long, RNA genome requires proofreading mechanisms. The nsp14/nsp10 subunits, along with the helicase nsp13, contribute to this process, ensuring transcription fidelity (Campagnola et al. 2022). Two nsp13 subunits in the RdRP complex bind to the helical structures of nsp8, with nsp13‐T and nsp13‐F acting from the thumb and finger domain sides of nsp12, respectively (Figure 4C). Interestingly, nsp13 is a processive helicase that unwinds dsRNA in the 5′→3′ direction of the template strand, opposite to the polymerase's 3′→5′ processing direction (Figure 4D). This raises questions about the specific function of nsp13 during processive transcription and its role in maintaining transcriptional fidelity. One possibility is that nsp13 induce RdRP backtracking, which seems to be a key mechanism for proofreading of eukaryotic RNA polymerases (Chen et al. 2020; Schwank et al. 2022). The RdRP backtracking mediated proofreading mechanism may substantially influence RdRP kinetics, leading to an extended duration of stalled RdRP on the template strand.

The detailed cryo‐EM study reveals different positions of nsp13‐T, dependent on the current state of the polymerase complex (Figure 4C) (Chen et al. 2020). The outlined stages span from the open state during polymerase transcription, through a swiveled state induced by nucleotide mispairing in the active site, to the closed state when the helicase unwinds the template–product dsRNA. The second helicase subunit, nsp13‐F, has not been demonstrated to bind RNA. Its presumed role is that of a steric factor, stabilizing various conformations of nsp13‐T. The helicase activity unzips the secondary structure in template RNA, inducing backtracking of the polymerase complex. This can result in the exposure of the 3′ end of the newly synthesized RNA to the nsp14/nsp10 exonuclease (Figure 4D) (Chen et al. 2022; Robson et al. 2020).

The structure of the nsp14 protein is highly flexible. The protein comprised two principal functional domains: the N‐terminal 3′–5′ exonuclease domain (ExoN) and the C‐terminal N7‐MTase domain, involved in the process of viral RNA capping. The exonuclease domain (ExoN) exhibits similarity to the DEDD exonuclease superfamily and is responsible for the excision of mismatched nucleotides from the nascent RNA strand (Bouvet et al. 2012). The binding of nsp10 cofactor stabilizes the structure of nsp14, placing the residues of the ExoN active site in the correct position to perform the hydrolysis reaction of the nucleotide from the RNA strand product (Bouvet et al. 2012; Ferron et al. 2018). The exact mechanism of action of the nsp14/nsp10 is not known. However, it is speculated that the mismatched nucleotide has to be exposed to the 3′–5′ exonuclease. The interaction of nsp14 with nsp12 polymerase indicates that nsp14/nsp10 acts as a part of RdRP holoenzyme (Bouvet et al. 2012).

### 4.3 RdRP Inhibitors

The search for drugs for the treatment of Covid‐19 includes potential RdRP inhibitors, with nucleoside analogs being a primary focus (Table 2). The adenosine analog remdesivir is introduced into the nascent RNA strand by RdRP and causes polymerase stalling (Gordon et al. 2020; Gordon et al. 2020). This “delayed chain termination” is caused by the steric clash between the remdesivir cyano group and Ser861 of nsp12 after the incorporation of the *n* + 3 nucleotide into the nascent RNA strand (Bravo et al. 2021; Kokic et al. 2021). Polymerase stalling caused by remdesivir can be overcome with high concentrations of ribonucleotides (Tchesnokov et al. 2020). This leads to “template‐dependent inhibition,” where remdesivir in the template strand blocks the elongation of the nascent strand.

The cytidine analog molnupiravir is incorporated into the nascent strand but does not cause RdRP stalling. Instead, it promotes mutation in the second round of gRNA replication (Kabinger et al. 2021). A similar mechanism of lethal mutagenesis is utilized by favipiravir, a purine analog (Shannon et al. 2020).

Nucleotide analogs that show antiviral effects against CoVs share a common feature of escaping the proofreading mechanism of the transcribing machinery. The inhibition of RdRP by remdesivir is suggested to result in impaired nsp14 exonuclease activity due to delayed RdRP stalling (Seifert et al. 2021). This, in turn, is thought to reduce the likelihood of nsp13‐induced backtracking and the exposure of the misincorporated nucleotide for removal. Furthermore, the bulky cyano group of remdesivir is postulated to prevent nucleotide excision by exonuclease. Molnupiravir has also been observed to evade the proofreading activity of the nsp14 exonuclease, though the specific mechanism remains unclear. It is hypothesized that the base pairing of molnupiravir with adenine or guanine is highly stable and does not induce the backtracking of RdRP required for nucleotide excision (Kabinger et al. 2021).

### 4.4 Auxiliary Functions of RdRP Complex

Beyond its polymerase activity, the nsp12 protein exhibits two additional functions mediated by the nidoviral RdRP‐associated nucleotidyltransferase (NiRAN) domain. The NiRAN domain facilitates the transfer of nucleoside monophosphate to the N terminus of the nsp9 protein (Schmidt et al. 2023; Wang, Svetlov, and Artsimovitch 2021). It was suggested that NMPylated nsp9 can serve as a protein primer for transcription. This hypothesis finds support in the observation that nsp9 binds to the 5′ end of both positive and negative strands of the SARS‐CoV‐2 genome (Schmidt et al. 2023). It is still not established if RdRP is a primer‐dependent polymerase. While earlier studies suggested nsp8 as an essential cofactor with a primase function (Imbert et al. 2006), subsequent research questioned this model (Tvarogová et al. 2019). Establishing an elongation–competent complex is a key energetic barrier in transcription, since only sufficiently long nascent RNA can support a stable elongation complex (Revyakin et al. 2006). Therefore, determining whether the RdRP is a primer‐dependent or primer‐independent polymerase is particularly relevant for understanding the initiation mechanism of CoV transcription.

Nsp12 also RNAylates nsp9, contributing to the viral mechanism of 5′ capping of newly synthesized RNA (Park et al. 2022). Previously, both the nsp16 and nsp14 proteins, possessing methyltransferase activity, along with their cofactor nsp10, were demonstrated to play a significant role in the viral mRNA capping process. The recently uncovered features of nsp9 suggest a novel and comprehensive mechanism for gRNA capping in CoVs. In this proposed mechanism, nsp9 and nsp12 contribute to cap core formation, while nsp14/nsp16 collaborate to transform the cap core into a mature mRNA (Park et al. 2022).

### 4.5 Host Cell Factors Involved in CoV Transcription

Several host cellular factors may be recruited to participate in viral genome transcription. The exploration of these factors focuses on viral RNA interactions with cellular proteins, and protein–protein interactions between virus and host factors.

#### 4.5.1 SND1

Recently, staphylococcal nuclease domain‐containing protein 1 (SND1) was identified as a regulator of SARS‐CoV‐2 transcription. As an RNA‐binding protein, SND1 appears essential for viral replication, binding to the negative strand of the viral genome and nsp9, thereby modulating nsp9 occupancy at the 3′ end of viral RNA. This finding introduces the first proposed mechanism by which a host factor regulates CoV transcription (Schmidt et al. 2023). Transcription from the negative strand is crucial for the dynamics of CoV infection and is discussed further in Section 5.4 of this review.

#### 4.5.2 hnRNP

Host proteins have been shown to bind specifically to the 5′ and 3’ UTR of viral RNA, selectively associating with either the positive or negative strand. These sequence‐specific interactions suggest a regulatory role for host proteins in viral RNA processes, including transcription. Members of the heterogeneous nuclear ribonucleoproteins (hnRNP) family, known for their roles in RNA processing and decay, have demonstrated affinity for CoV RNA. Notably, hnRNP A1 has been shown to bind to the 3′ end of the mouse hepatitis virus (MHV) negative strand, positively implicating it in subgenomic RNA transcription (Li et al. 1997; Zhang and Lai 1995). Further studies suggest that other hnRNP A/B family members may compensate for hnRNP A1 deficiency in cells lacking its expression (Shi, Yu, and Lai 2003), suggesting redundancy of hnRNP in CoV transcription.

Another key regulator is the polypyrimidine tract‐binding protein (PTB), also known as hnRNP I, which binds to the leader sequence of MHV plus‐strand RNA. Given hnRNP A1's interaction with the negative strand leader sequence and intergenomic region, it is proposed that these host proteins may cooperate in viral discontinuous transcription (Li et al. 1999). Additionally, synaptotagmin‐binding cytoplasmic RNA‐interacting protein (SYNCRIP), another hnRNP family member, binds to the 5′ end of both RNA strands and is critical for MHV RNA synthesis, as its downregulation reduces viral RNA production (Choi, Mizutani, and Lai 2004). While these specific interactions have been confirmed in vivo, the detailed mechanisms through which hnRNP proteins influence CoV transcription remain unclear.

#### 4.5.3 Other Factors

Following the SARS‐CoV‐2 pandemic, interest in CoV molecular mechanisms surged, leading to comprehensive proteomic studies that have identified extensive interactions between viral and host proteins. These studies highlight the regulation of cellular pathways during CoV infection and identify potential host protein candidates that could act as viral protein cofactors (Chen et al. 2021; Gordon et al. 2020; Stukalov et al. 2021). Notably, human splicing factors SLU7, PPIL3, and AKAP8 have been shown to interact with nsp12, positively influencing SARS‐CoV‐2 RdRP stability and viral replication in vivo. While these factors are suggested to contribute to RdRP complex assembly, this role was not confirmed in the in vitro transcription assays. Instead, interaction of nsp12 with these splicing factors appears to contribute to the alternative splicing of human mRNAs (Yang et al. 2024).

## 5 Kinetics of RDRP Activity

The outlined viral life cycle may suggest a linear order of events, whereas actually some stages of infection have to occur sequentially and other simultaneously, what may raise conflicts especially in the early hours. Translation and transcription initially work on the same ssRNA(+) molecule, with translation of several proteins needed to form the RdRP. Understanding the kinetics of these events brings us closer to identifying regulatory mechanisms.

**FIGURE 5:**
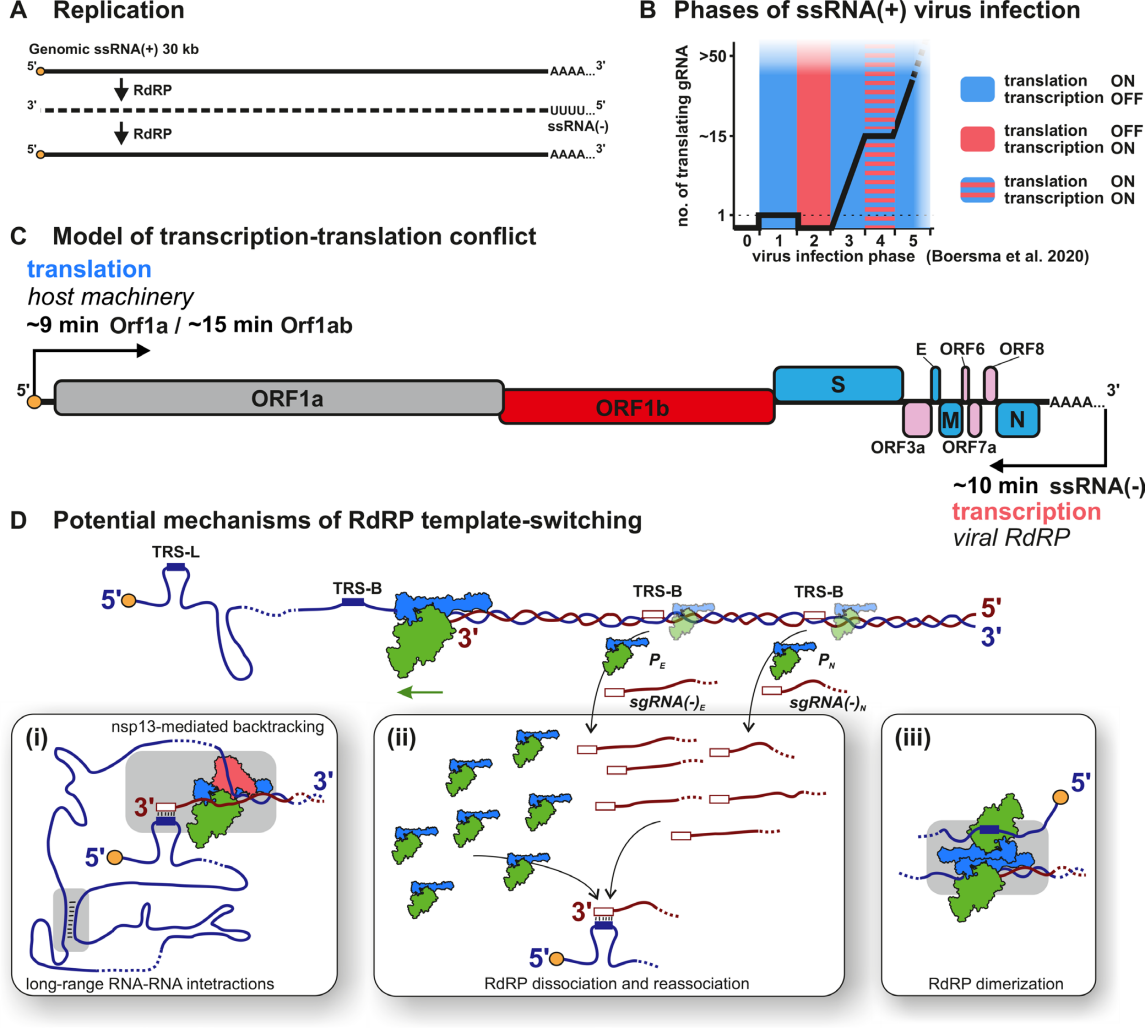
Kinetics of RdRP activity (on the basis of Boersma et al. 2020; Jochheim et al. 2021; Malone et al. 2021; Sawicki and Sawicki 1998). (A) Replication of SARS‐CoV‐2 genome requires transcription of ssRNA(−) serving further as a template for the synthesis of genomic ssRNA(+). (B) Phases of ssRNA(+) infection as per Boersma et al. (2020). Experimental results showed the viral translation dynamics (black line), interpreted considering steric conflicts between transcription and translation machinery (colored background). Detailed description in text. (C) Model outlining conflict between translation and transcription during the very first round of viral ssRNA(+) replication. Viral transcription machinery (RdRP) is translated from ORF1b (red), therefore ssRNA(+) has to be cleared from ribosomes to allow RNA transcription by RdRP. (D) Potential mechanisms behind discontinuous transcription. In this process, the RdRP skips over substantial stretches of the genome to link the 5′ leader sequence with downstream segments, creating distinct sgRNAs with given probability *p*. Potential mechanisms include: (i) long‐range RNA–RNA interactions and nsp13‐induced backtracking of RdRP, (ii) dissociation and reassociation of RdRP, and (iii) RdRP dimerization. These mechanisms are not mutually exclusive.

Transcription results in the production of new RNA as double‐stranded RNA, accompanied by the template RNA, which could activate pattern recognition receptors like MDA5 and initiate antiviral signaling pathways (Chen and Hur 2022). This makes the virus particularly susceptible to cellular defense mechanisms during the initial hours of its life cycle. Consequently, the timing of transcriptional processes is critical for the success or failure of CoV infection, especially since two rounds of 30‐kb‐long, full‐length transcription are required to produce a copy of the gRNA (Figure 5A).

### 5.1 Translation Precedes Transcription

In CoVs, the same ssRNA(+) molecule serves as both the genome and mRNA. The viral transcription machinery, along with other nonstructural proteins, is translated from two open reading frames (ORFs). The catalytic subunit of the RdRP is translated from ORF1b, which requires ribosomal frameshifting—a process that occurs in a fraction of events (Nakagawa, Lokugamage, and Makino 2016). This unique arrangement results in significant variability in the expression levels of CoV proteins within infected cells. Products encoded by ORF1b, including the main replication machinery components nsp12 and nsp13, tend to have very low abundance (Finkel et al. 2021). Consequently, the formation of the very first RdRP complex depends on the successful translation of several proteins beforehand.

This observation has important implications for the kinetics of viral transcription. Assuming an average translation rate of eight amino acids per second (Li, Bickel, and Biggin 2014), the first functional RdRP molecule could be available about 15 min after successful translation initiation on ssRNA(+), but RdRP assembly and initiation via nsp9 might take longer. Due to frameshifting probability, the number of RdRP molecules will be 5‐ to 10‐fold lower than proteins translated from ORF1a (nsp1–nsp11). The accumulation of nsp1–11 may play a role in suppressing the cellular antiviral response and safeguarding the 30‐kb‐long gRNA.

The before consideration could be significantly affected by the presence of RdRP delivered with gRNA in the viral envelope. The mass of viral gRNA is estimated at 10.2 MDa, not including N protein molecules, while the RdRP core is only 0.16 MDa, making its size negligible for the entire virion size. Although there are no data on the mechanism of packaging RdRP into virions, it cannot be excluded that some virions carry RdRP molecules.

### 5.2 RdRP‐Directed Replication and Transcription–Translation Conflict

Replication of ssRNA(+) viruses involves continuous synthesis of ssRNA(−) to create a full‐length complementary template strand that will be copied into multiple ssRNA(+) (Robson et al. 2020). However, ongoing translation of ssRNA(+) raises a steric conflict between the translation and transcription machinery. Translation of ORF1ab follows the 5′–3′ direction of ssRNA(+), whereas replication proceeds in the opposite direction. Both processes require continuous contact with the ssRNA(+) threaded through each particle's template channel (Figure 5B,C).

One might expect that a translating ribosome could displace or even strip a transcribing RdRP. Ribosomes are molecular machines that utilize four GTP molecules for each translocation step, moving three nucleotides at a time (Noller et al. 2017). In contrast, transcription is a Brownian ratchet mechanism, where translocation is bidirectional and nucleotide addition provides directionality (Noe Gonzalez, Blears, and Svejstrup 2021; Turowski et al. 2020). The RNA polymerase backtracking allows for the nascent transcript proofreading. Due to these differences, it remains unlikely that RdRP can overcome a translating ribosome. It is very likely that the ribosome would cause the RdRP to stop, backtrack, or even be stripped by the translation machinery.

Kinetics must relate to RdRP speed. Biochemical studies on SARS‐CoV‐2 have reported various RdRP elongation rates, such as 100 nt/s and 200 nt/s, using core subunits (Figure 4A). These high elongation rates have not been observed in cells with long products and fully processive RdRP. It should be noted that under cellular conditions, the availability of nucleotides is limited, the RNA template is coated with proteins, processive transcription is maintained for thousands of nucleotides, and RdRP exhibits proofreading activity. All these factors influence the transcription elongation rate, making the velocity of other RNA polymerases measured in vivo more relevant. Therefore, for our considerations, we use the average transcription elongation rate in vivo, which is 40–80 nt/s (Turowski et al. 2020). Given the large size of the SARS‐CoV‐2 genome, RdRP transcription would take approximately 10 min. Newly synthesized ssRNA(−) pairs with ssRNA(+), and dsRNA is known to induce host immune responses (Gantier and Williams 2007), suggesting the presence of mechanisms that protect the viral genome during the switch from translation to transcription of the first ssRNA(−).

Single‐molecule investigations of picornaviruses, another group of ssRNA(+) viruses, have shed light on the translation kinetics by directly observing the translation of SunTagged viral proteins (Boersma et al. 2020). These studies identified five distinct phases of viral infection, each corresponding to different stages of translation. We used these phases to annotate transcriptional activity (Figure 5B) and predict that in phase 2, translation is repressed to allow for the very first round of ssRNA(−) synthesis. It is important to note that due to the shorter length of the constructs used in this research, the duration of each phase is not precise, but provides a general understanding of ssRNA(+) virus kinetics.

Therefore, how is the first round of processive, 30‐kb‐long transcription achieved? Nsp1, encoded by ORF1a, plays a major role in translation inhibition by blocking the mRNA entry tunnel in the ribosome (Lapointe et al. 2021; Thoms et al. 2020). Temporary inhibition of translation near transcribing RdRP would be a potential mechanism. However, the 5’ UTR of SARS‐CoV‐2 contains the stem‐loop 1 (SL1) region, which evades nsp1‐mediated translational suppression (Vora et al. 2022) and it remains unknown if a high nsp1 dosage effect would be enough to overcome SL1 effect. Another possibility is the formation of replication organelles, specialized compartments that segregate viral RNA synthesis from cellular processes.

### 5.3 Discontinuous Transcription of sgRNAs

sgRNAs are composed of elements present in distant locations within the genomic ssRNA(+): the 69‐nt‐long 5′ leader sequence and the 3′ end of different lengths. To synthesize sgRNAs, CoVs use discontinuous transcription, where RdRP “jumps” over large genome regions before resuming transcription (Enjuanes et al. 2006; Fung and Liu 2019; Sola et al. 2015). the “jump” mechanism is also called template switching or strand switching.

A commonly accepted model proposes that template ssRNA(+) undergoes discontinuous transcription to sgRNA(−), and subsequent amplification yields high copy numbers of sgRNA(+) mRNAs (Enjuanes et al. 2006; Fung and Liu 2019; Sawicki and Sawicki 1998). The exact mechanism of template switching remains unknown (Figure 5D), but new structural data reveal long‐range RNA–RNA interactions potentially involved in this process (Huston et al. 2021; Zhang et al. 2021) (Figure 5D, panel i). The demonstration of the dimeric form of SARS‐CoV‐2 RdRP suggested an intriguing possibility that the “jump” might be enabled through direct interaction between two RdRP molecules (Jochheim et al. 2021) (Figure 5D, panel iii).

Another explanation could be based on thermodynamic characteristics of elongating polymerases. Mechanistically, this would involve the dissociation (similar to premature termination) and reassociation of nascent transcript with the template strand through TRS‐B–TRS‐L interaction (see Figure 5D, panel ii). This mechanism is supported by spontaneous binding of DNA‐ and RNA‐dependent polymerase complexes to the template in vitro (Kohler et al. 2017; Kuhn et al. 2007; Turowski et al. 2020), including the viral RdRP (Hillen et al. 2020). Additionally, RNA polymerase II in mammalian genomes initiates transcription in vivo using RNA:DNA hybrids between RNA and template DNA strand (Tan‐Wong, Dhir, and Proudfoot 2019).

The mechanism of discontinuous transcription regulates the stoichiometry between structural proteins. Each sgRNA(+) contains a common 5′ leader sequence followed by the ORF with its stop codon. The remaining ORFs function as the 3’ UTR sequence. Abundance of sgRNA(−) reflects levels of sgRNA(+), meaning “jump” probability at given TRS‐B impacts sgRNA transcription rate (Figure 5D). This is supported by data measuring SARS‐CoV‐2 sgRNA and protein abundance (Finkel et al. 2021; Kim et al. 2020), showing higher expression levels for 3′ end proximal genes.

### 5.4 Template RNA Assignment to Available RdRP

CoV infection leads to the rapid synthesis of viral RNA, with up to 90% of cellular RNA being viral after several hours of infection (Schmidt et al. 2021). Given the high number of viral RNA copies, including ssRNA(+), ssRNA(−), sgRNA(+), and sgRNA(−), a critical question arises: how does the viral RNA‐dependent RNA polymerase (RdRP) select the appropriate template strand for replication?

This selection process is further complicated by the fact that the 3′ ends of ssRNA(+) contain poly(A) tails, which are similar to the cellular mRNAs. The presence of these poly(A) tails necessitates a specific mechanism by which RdRP can distinguish between viral and host RNA. The selection of template RNA by RdRP is a vital aspect of CoV replication that involves intricate interactions between viral and host factors. These interactions determine the efficiency and fidelity of viral RNA synthesis, making this process crucial to CoVs ability to replicate its RNA.

## 6 Conclusion

Understanding the biology of viruses reveals a fascinating mixture of common biological mechanisms and unique virus‐specific features. To comprehend how coronaviruses (CoVs) reprogram host cells, it is essential to examine both viral factors and their interactions with host factors. Focusing on viral transcription, we outline how host elements, such as ribosomes and viral components like the RdRP depend on each other, adding temporal layer of complexity to virological studies. This raises several unresolved questions about CoV biology that require further investigation:

- Does the synthesis of initial ssRNA(−) depend on DMV formation?
- Can functional RdRP molecules be transported within a CoV virion?
- What mechanism inhibits translation initiation to permit the first round of ssRNA(−) synthesis?
- Which mechanism is responsible for template‐switching (Figure 5D)?
- How does RdRP select the template RNA strand?

We believe that considering kinetic factors will help to identify new regulatory mechanisms in viral biology. This understanding will be crucial for developing novel antiviral drugs and treatment strategies, ultimately aiding in the fight against CoV infections.

## Author Contributions

**Katarzyna Grelewska‐Nowotko:** conceptualization (equal), investigation (equal), validation (equal), visualization (equal), writing – original draft (lead), writing – review and editing (lead). **Ahmed Eisa Elhag:** conceptualization (supporting), investigation (supporting), visualization (supporting), writing – original draft (equal), writing – review and editing (supporting). **Tomasz Wojciech Turowski:** conceptualization (lead), funding acquisition (lead), investigation (supporting), supervision (lead), validation (equal), visualization (lead), writing – original draft (equal), writing – review and editing (equal).

## Conflicts of Interest

The authors declare no conflicts of interest.

## Related WIREs Articles

Epitranscriptomic marks: Emerging modulators of RNA virus gene expression

Emerging translation strategies during virus‐host interaction

## Data Availability

Data sharing is not applicable to this article as no new data were created or analyzed in this study.
